# Cerebrospinal fluid lipoprotein-mediated cholesterol delivery to neurons is impaired in Alzheimer's disease and involves APOE4

**DOI:** 10.1016/j.jlr.2025.100865

**Published:** 2025-07-21

**Authors:** Carla Borràs, Marina Canyelles, David Santos, Noemí Rotllan, Estefanía Núñez, Jesús Vázquez, Daniel Maspoch, Mary Cano-Sarabia, Qi Zhao, Maria Carmona-Iragui, Sònia Sirisi, Alberto Lleó, Juan Fortea, Daniel Alcolea, Francisco Blanco-Vaca, Joan Carles Escolà-Gil, Mireia Tondo

**Affiliations:** 1 Institut de Recerca Sant Pau (IR SANT PAU), Barcelona, Spain; 2 Departament de Bioquímica i Biologia Molecular, Universitat Autònoma de Barcelona, Barcelona, Spain; 3 CIBER de Diabetes y Enfermedades Metabólicas Asociadas, CIBERDEM, Madrid, Spain; 4 Department of Biochemistry, Hospital de la Santa Creu i Sant Pau, Barcelona, Spain; 5 Centro Nacional de Investigaciones Cardiovasculares Carlos III, Madrid, Spain; 6 CIBER de Enfermedades Cardiovasculares, CIBERCV, Madrid, Spain; 7 Catalan Institute of Nanoscience and Nanotechnology (ICN2), CSIC and The Barcelona Institute of Science and Technology, Barcelona, Spain; 8 Departament de Química, Facultat de Ciències, Universitat Autònoma de Barcelona (UAB), Barcelona, Spain; 9 ICREA (Institució Catalana d’Investigació i Estudis Avançats), Barcelona, Spain; 10 Sant Pau Memory Unit, Department of Neurology, Hospital de la Santa Creu i Sant Pau, Barcelona, Spain; 11 CIBER de Enfermedades Neurodegenerativas, CIBERNED, Madrid, Spain; 12 Barcelona Down Medical Center, Fundació Catalana de Síndrome de Down, Barcelona, Spain

**Keywords:** Alzheimer's disease, APOE, cholesterol, CSF lipoprotein

## Abstract

In the central nervous system, apolipoprotein (APO)E-containing lipoprotein particles mediate the transport of glial-derived cholesterol to neurons, which is essential for neuronal membrane remodeling and maintenance of the myelin sheath. We aimed to examine cholesterol transport via lipoprotein particles in cerebrospinal fluid (CSF) of Alzheimer’s disease (AD) patients compared to control individuals. Additionally, we explored the ability of reconstituted HDL containing different APOE isoforms to regulate cholesterol transport. We evaluated the capacity of CSF lipoprotein particles to facilitate radiolabeled unesterified cholesterol efflux from A172 human glioblastoma astrocytes and to deliver cholesterol to SH-SY5Y human neuronal cells. The CSF lipoprotein proteome was analyzed by LC-MS/MS. Reconstituted HDL nanoparticles were prepared by combining phospholipids and cholesterol with human APOE3 or APOE4, followed by radiolabeling with unesterified cholesterol. Our results showed that cholesterol efflux from astrocytes to CSF were similar between AD patients and controls, both under baseline conditions and after activation of ABCA1 and ABCG1. However, CSF lipoprotein-mediated neuronal cholesterol uptake was significantly reduced in the AD group. LC-MS/MS analysis identified 239 proteins associated with CSF lipoproteins in both groups, with no major alterations in proteins linked to cholesterol metabolism. However, 27 proteins involved in noncholesterol-related processes were differentially expressed. Notably, synthetic reconstituted HDL particles containing APOE4 exhibited reduced capacity to deliver cholesterol to neurons compared to those with APOE3. These findings indicate that CSF lipoproteins from patients with AD demonstrate impaired cholesterol delivery to neurons. Our study highlights APOE4 as a critical contributor to abnormal neuronal cholesterol uptake in AD pathophysiology.

Alzheimer’s disease (AD) is a neurodegenerative disorder that causes difficulty communicating and reasoning, mood changes, and progressive memory loss. Histologically, AD is defined by the pathologic accumulation of extracellular amyloid beta (Aβ) and abnormally hyperphosphorylated intracellular tau filaments in neurons, resulting in amyloid plaques and neurofibrillary tangles, respectively, with neuropathological lesions occurring many years before clinical signs (1). With few effective and approved treatments, AD is a growing public health concern.

The brain is predominantly composed of lipids (2), which are essential for functions such as blood-brain barrier regulation, amyloid precursor protein processing, myelination, membrane remodeling, receptor signaling, oxidation, inflammation, and energy balance (3). Cholesterol, in particular, is vital for brain health, neuron repair, membrane remodeling, and plasticity (4), and its metabolism has been extensively implicated in the pathogenesis of AD (5, 6, 7).

Since mature lipoproteins containing cholesterol cannot cross the blood-brain barrier, cholesterol synthesis in the central nervous system (CNS) primarily relies on astrocytes (8). Cerebrospinal fluid (CSF), the most nearby biofluid to the brain that can be used to evaluate both normal and aberrant brain physiology, contains unique lipoproteins, also known as "high density lipoprotein (HDL)-like particles" due to their similarity to peripheral HDL. These particles are present at concentrations 100 times lower than plasma HDL (9), making comprehensive biochemical analysis highly challenging. CSF lipoproteins are essential for cholesterol transport between glial cells and neurons, with apolipoprotein (APO)E as the primary protein necessary for this process (8). APOE must be synthesized locally by astrocytes and microglia (8). Notably, lipidated APOE contributes to the clearance of Aβ peptides, thus potentially impacting their transport in the CNS and contributing to AD pathophysiology (10, 11). In this context, recent research has confirmed that APOE4 homozygosity is a significant genetic factor contributing to AD (12). Disruption of cholesterol homeostasis has been increasingly implicated in AD, particularly in the context of APOE4-related lipid trafficking defects. Although direct evidence for neuronal cholesterol depletion in AD brains remains limited, studies suggest that APOE4 impairs lipid delivery to neurons, potentially compromising synaptic integrity and accelerating neurodegeneration (13, 14, 15). Regarding cholesterol efflux to CSF lipoproteins in the CNS, ABCA1 is the main cholesterol efflux transporter in astrocytes, with ABCG1 playing a secondary role (16, 17). Cholesterol efflux to the CSF is a potential mechanism in AD-related cholesterol metabolism deregulation (18, 19), yet studies using human astrocytes to examine this in AD patients' CSF are lacking. Concerning cholesterol uptake, CSF lipoproteins deliver cholesterol to neurons through APOE interaction with specific lipoprotein receptors such as LDL receptor (LDLR), LDLR-related protein 1 (LRP1), very-low density lipoprotein receptor, and APOE receptor 2 (20). In this regard, proprotein convertase subtilisin/kexin type 9 (PCSK9) has been suggested as a potential player, since it degrades some APOE-binding receptors (21).

In this study, we investigated CSF-mediated cholesterol transport between human glioblastoma astrocytes and neurons via CSF lipoproteins from control individuals and AD patients, examining its association with AD biomarkers and the CSF lipoprotein proteome. In addition, we developed synthetic reconstituted HDL (rHDL) nanoparticles containing either APOE3 or APOE4 to assess the impact of the APOE-ε4 isoform on cholesterol transport. Our findings reveal that CSF lipoproteins from AD patients show reduced efficiency in delivering cholesterol to neurons, with APOE4 emerging as a potential key factor in the disrupted neuronal cholesterol uptake observed in AD pathophysiology.

## Materials and Methods

### Human samples

CSF samples were retrospectively selected from the SPIN cohort (Sant Pau Initiative on Neurodegeneration), a multimodal research cohort for biomarker discovery and validation that includes participants with different neurodegenerative dementias, mild cognitive impairment, and cognitively normal controls. All participants underwent an extensive neurological and neuropsychological evaluation and had blood extraction and lumbar puncture for CSF AD biomarker analysis as part of their diagnostic work-up (22). All participants provided written consent to participate in our biomarker program. Further information on the SPIN cohort can be found at https://santpaumemoryunit.com/our-research/spin-cohort.

A total of 20 CSF from control individuals (n = 10) and patients with AD dementia (n = 10) were included. CSF samples were obtained by lumbar puncture under standardized conditions (22). All participants in both the healthy control and disease groups were White and of Caucasian descent. To minimize potential blood contamination, the first 1–2 ml of CSF was discarded during collection, and all samples were visually inspected to ensure clarity and absence of red blood cells or turbidity. Samples were then centrifuged at 2,000 g for 10 min, and only the supernatant was used for analysis (22). To further exclude plasma contamination, APOB levels were measured in pooled CSF samples using an immunoturbidimetric assay on the COBAS 6000/501 platform; APOB was consistently undetectable, confirming the absence of plasma-derived lipoproteins.

All participants from the SPIN cohort provided written informed consent for the acquisition, analysis, and storage of biological samples. They were explicitly informed about the potential use of anonymized data and/or biological samples for research purposes. The study was approved by the Sant Pau Ethics Committee (Protocol code: EC/22/202/6880) following the standards for medical research in humans recommended by the Declaration of Helsinki.

### CSF AD biomarker analysis

APOE genotype, defined by two single nucleotide polymorphisms, the rs429358 and the rs7412 was performed by Sanger sequencing as previously reported (22). Brain amyloidosis biomarkers (CSF Aβ_1-40_, CSF Aβ_1-42_, and CSF Aβ_42_/Aβ_40_ ratio), tau pathology biomarkers (CSF phosphorylated-Tau181 (p-Tau)) and neurodegeneration biomarkers (CSF total-Tau (t-Tau)) were determined by chemiluminescent immunoassay on a LUMIPULSE G600II automated platform analyzer (Fujirebio®).

### CSF lipid-related parameters measurement

Total CSF cholesterol and free cholesterol were measured by Amplex Red Cholesterol Assay Kit (Thermo Fisher Scientific). APOE, APOJ, and APOA1 concentrations were measured by Human ELISA Kits (Thermo Fisher Scientific). PCSK9 concentrations were determined using the Quantikine® ELISA kit (R&D Systems). The kits were designed for plasma samples quantification; therefore, CSF samples dilutions were adapted to the cholesterol, APOs, or PCSK9 concentrations in the CSF.

### Cell culture

Human glioblastoma cells A172 (ATCC® CRL-1620™) were maintained in DMEM high glucose with L-glutamine and with sodium pyruvate (Corning) supplemented with 10% FBS (Pan Biotech) and 100 U/ml penicillin/streptomycin (Dominique Dutscher). Human neuroblastoma cells SH-SY5Y (ATCC® CRL-2266™) were maintained in DMEM high glucose with L-glutamine (Corning) and Ham's Nutrient Mixture F12 with L-glutamine (Cytiva) 1:1 v/v supplemented with 10% FBS (Pan Biotech) and 100 U/ml penicillin/streptomycin (Dominique Dutscher). Cells were seeded and grown in 75 cm^2^ cell culture flasks and incubated in a humidified incubator (5% CO_2_, 37°C). The medium was renewed every 48 h, and cells were trypsinized once they reached confluence.

Previous to any experiment, human neuroblastoma SH-SY5Y cells were differentiated into functional neurons by replacing maintenance medium with differentiation culture medium for 7 days and refreshment every 72 h. Differentiation culture medium consisted of DMEM high glucose with L-glutamine (Corning) and Ham's Nutrient Mixture F12 with L-glutamine (Cytiva) 1:1 v/v supplemented with 1% FBS, 100 U/ml penicillin/streptomycin, and 10 μM retinoic acid (Sigma-Aldrich/Merck).

### Cholesterol efflux assay

A172 and SH-SY5Y cells were seeded at densities of 50,000 and 100,000 cells per well, respectively, in 24-well plates using their corresponding maintenance culture medium. SH-SY5Y cells were differentiated by incubating them in differentiation culture medium containing retinoic acid, as previously described. Twenty-four hours after plating or reaching differentiation, cells were loaded with 0.5 μCi/well of radiolabeled cholesterol ([1α,2α(n)-^3^H] cholesterol, Revvity) added to 5% FBS-supplemented medium. Cells were allowed to capture radiolabeled cholesterol for 48 h and extensively washed with PBS before performing cholesterol efflux assays. Subsequently, the cells were equilibrated overnight in serum-free medium containing 0.2% free fatty acid BSA (Sigma-Aldrich/Merck), either with or without the addition of the ABCA1/G1 activator T0901317 (Cayman Chemical) at a concentration of 2 μM. Following this, cells were washed and serum-free medium containing either CSF (30% v/v), lipid-free APOA1 (20 μg/ml), lipid-free APOE (20 μg/ml), or rHDL (5 μg of APOE/ml) was added to the cells. After 4 h, the medium was collected and 0.1 M NaOH (Sigma-Aldrich/Merck) was added to the cells. The medium fraction was centrifuged for 5 min at 250 g to remove floating cells. The cellular fraction was incubated at 4°C with gentle shaking for 48 h, after which it was collected and sonicated for at least 1 h. Radiolabeled cholesterol in both medium and cellular fraction was quantified by liquid scintillation counting, and the percentage of cholesterol efflux was calculated by dividing radiolabeled cholesterol in the medium by the sum of radiolabeled cholesterol in the medium and cellular fractions.

### Cholesterol uptake assay

CSF samples or synthetic rHDL nanoparticles were radiolabeled with evaporated [1α,2α(n)-^3^H] cholesterol (0.1 mCi/ml and 0.2 mCi/ml respectively) by incubating them overnight at 37°C. The incorporation of radiolabeled cholesterol into lipoproteins of CSF was verified by isolating lipoprotein particles at a density range of 1.063–1.210 g/ml using density gradient ultracentrifugation. A172 and SH-SY5Y cells were seeded at a density of 100,000 cells per well in 24-well plates. Twenty-four hours after plating or reaching differentiation, serum-free medium containing radiolabeled CSF (10% v/v) or rHDL (5 μg of APOE/ml) was added to the cells. Part of the experiments was conducted in the presence of Human Tau-441/2N4R Protein (ACROBiosystems) or Aβ Protein Fragment _1-42_ (Sigma-Aldrich/Merck), which were added to the medium at varying concentrations. After 4 h, the medium was collected and 0.1 M NaOH (Sigma-Aldrich/Merck) was added to the cells. The medium fraction was centrifuged for 5 min at 250g to remove floating cells. The cellular fraction was incubated at 4°C with gentle shaking for 48 h. Afterward, it was collected and sonicated for at least 1 h. Radiolabeled cholesterol in both the medium and cellular fraction was quantified by liquid scintillation counting, and the percentage of cholesterol uptake was calculated by dividing radiolabeled cholesterol in the cellular fraction by the sum of radiolabeled cholesterol in the medium and the cellular fraction. Values were normalized to the cellular protein content, determined using the Pierce™ BCA Protein Assay Kit (Thermo Fisher Scientific).

### Quantitative Real Time-PCR

For quantitative Real Time-PCR analyses, A172 and SH-SY5Y cells were seeded at a density of 150,000 cells per well in 12-well plates and cholesterol efflux steps were mimicked. Afterward, RNA was isolated using the EZ-10 DNAaway RNA Miniprep kit (Bio Basic) and quantified by NanoDrop-2000 spectrophotometry (Thermo Fisher Scientific). Complementary DNA was generated using EasyScript First-Strand cDNA Synthesis SuperMix (Transgen Biotech) and quantitative Real-Time PCR amplification was performed using the GoTaq(R) Probe qPCR Master Mix (Promega). Specific TaqMan probes (Applied Biosystems) were used for *ABCA1* (Hs01059118_m1), *ABCG1* (Hs00245154_m1), and *GAPDH* (Hs02758991_g1), the latter used as internal control gene. Reactions were run on a CFX96^TM^ Real-Time System (Bio-Rad) according to the manufacturer’s instructions. Thermal cycling conditions included 10 min at 95°C before the onset of the PCR cycles, which consisted of 40 cycles at 95°C for 15 s, and at 65°C for 1 min. The relative mRNA expression levels were calculated using the ΔΔCt method (23).

### Protein staining and blotting

Plasma HDL was isolated by ultracentrifugation at a density range of 1.063–1.210 g/ml, and the APOA1 concentration was determined using an immunoturbidimetric assay adapted for the COBAS 6000/501c autoanalyzer (Roche Diagnostics). Samples containing plasma HDL, lipid-free APOA1, APOE3, APOE4 (all from Sigma-Aldrich/Merck), or CSF were prepared by adding 40% v/v of 50% m/v sucrose (Sigma-Aldrich/Merck) to increase sample density and ensure proper loading into the wells during native agarose gel electrophoresis. Subsequently, 30 μl of each sample was size-separated electrophoretically on 4%–15% Mini-PROTEAN® TGX™ Precast Protein Gels (Bio-Rad) at 80 V for 30 min, followed by 120 V for 2 h. Precision Plus Protein™ All Blue Prestained Protein Standards (Bio-Rad) was used as molecular weight marker.

For protein staining, a fixation solution consisting of 40% methanol (Sigma-Aldrich/Merck) and 10% acetic acid (Sigma-Aldrich/Merck) was applied to the gels for 1 h. Gels were then washed with water and stained overnight with GelCode™ Blue Stain Reagent (Thermo Fisher Scientific). The following day, the gels were washed again with water to remove background staining.

For blotting, proteins were transferred onto 0.2 μm PVDF membranes (Bio-Rad). The membranes were blocked using EveryBlot Blocking Buffer (Bio-Rad) for 15 min and incubated overnight at 4°C with the goat anti-human APOE primary antibody (1:500 dilution, Roche Diagnostics). After incubation, the membranes were washed three times for 10 min each with TBS containing 0.1% of Tween-20 buffer and subsequently incubated with a rabbit anti-Goat IgG HRP-conjugated secondary antibody (Thermo Fisher Scientific) for 1 h. The membranes were washed three times each for 10 min with TBS containing 0.1% of Tween-20 buffer and analyzed using Clarity Western ECL Substrate (Bio-Rad).

Images were captured using a ChemiDoc XRS Gel Documentation System (Bio-Rad) and Image Lab software (version 6.0.1, Bio-Rad).

### Proteomic analysis

For the proteomic experiments, we used 30 μl of nonconcentrated CSF, which consisted of 25 μl of native CSF mixed with 15 μl of 50% sucrose solution to aid in sample loading. CSF samples were size-separated electrophoretically on 4%–15% Mini-PROTEAN® TGX™ Precast Protein Gels (Bio-Rad) as described above. After staining, CSF lipoprotein bands known to contain APOE from blotting analyses were excised. Each band was placed in an individual tube with acetonitrile (Sigma-Aldrich/Merck) which was removed under vacuum. The bands were stored at −80°C until further analysis.

Bands were rehydrated with 1 ml of bicarbonate 25 mM pH 8.8 and dehydrated with acetonitrile 100%. Protein denaturation was performed incubating the bands with 400 μl of 10 mM DL-dithiothreitol in 25 mM bicarbonate pH 8.8 for 30 min at room temperature and rotation. For protein alkylation, bands were incubated with 400 μl of 54 mM iodoacetamide in 25 mM bicarbonate pH 8.8 for 45 min at room temperature in rotation and protected from the light. Bands were dehydrated with acetonitrile 100% for 10 min and dried in the speed-vacuum. Protein digestion was performed with 1 μg of trypsin per band in 50 mM bicarbonate pH 8.8 and acetonitrile 10% buffer overnight at 37°C. Samples were desalted using NestGroup Spin columns following the manufacturer’s instructions, dried in the speed-vacuum and stored at 4°C until LC-MS/MS analysis.

LC-MS/MS analysis was conducted using an Evosep One HPLC (Evosep) coupled to an Orbitrap Eclipse Tribrid Mass Spectrometer (Thermo Fisher Scientific) using an Evotip C18 (Evosep) as trapping matrix and an Endurance Evosep (EV1106) column (15 cm × 150 μm ID) coupled to a stainless emitter of 30 μm ID and interfaced with the mass spectrometer using Nanospray Flex Ion Source. Column was heated to maintain temperature at 55°C. Peptides were eluted from Evotips and analyzed using Evosep One pre-programmed gradient for 15 samples per day. Samples were analyzed using a data independent acquisition strategy. Full MS resolutions were set to 120,000 at m/z 200 and the full MS AGC target was 300% with a maximum injection time set to Auto. The AGC target value for fragment spectra was set to 1,000%. Fifty windows of 12 m/z scanning from 400 to 100 m/z were employed with an overlap of 1 Da. MS2 resolution was set to 30,000, IT to 54 ms, and normalized collision energy to 30%. Raw files were analyzed with DIANN (version 1.8.1) using an in silico predicted spectral library derived from *Homo sapiens* proteome Uniprot Database (2022 version, containing 20.958 entries). N terminal excision of methionine and carbamidomethylation of cysteine were set as fixed modifications, while oxidation of methionine was set as a variable modification. The enzyme/cleavage rule was set to Trypsin/P, the digestion type was specific, and a maximum of one missed cleavage per peptide was allowed. Normalization was turned off, and quantification was conducted using Robust LC.

Quantitative information obtained from precursor intensities, as reported by DIANN, was integrated from the precursor level to the peptide level, and then to the protein level, using the WSPP model and Generic Integration Algorithm (24, 25), implemented through the iSanXot program (26, 27). In this model, quantitative protein values are expressed using the standardized variable Zq (normalized log2-ratios expressed in units of standard deviation based on the estimated variances). For functional analysis, proteins were annotated using DAVID (28). The analysis included proteins consistently detected across all samples and quantified with more than one peptide, with the full set of quantified proteins in the experiment used as background. Functional categories with an EASE score lower than 0.05 were considered significantly enriched.

### Preparation and characterization of synthetic reconstituted APOE3-and APOE4-containing lipoproteins

Synthetic rHDL particles containing APOE3 or APOE4 (rHDL-APOE3 and rHDL-APOE4) were generated using the cholate-dialysis method (29). 1,2-Dimyristoyl-sn-glycero-3-phosphocoline (DMPC, Merck KGaA) was selected as the phospholipid for rHDL particle reconstitution due to its well-defined biophysical properties, including a phase transition temperature near room temperature (∼24°C), which enables controlled thermal cycling during HDL assembly. Its saturated fatty acid chains form tightly packed, ordered bilayers, providing structural consistency and reproducibility in APOE-based rHDL preparations. Briefly, the lipid mixture was prepared with DMPC and free cholesterol (Merck KGaA) in a chloroform solution at a 9:1 DPMC/free cholesterol molar ratio. Synthetic rHDL-APOE3 and rHDL-APOE4 nanoparticles were fluorescently labeled for specific experiments. To this end, Oregon Green™ 488 1,2-Dihexadecanoyl-sn-Glycero-3-Phosphoethanolamine (Oregon Green™ 488 DHPE, Thermo Fisher Scientific) was added into the lipid mixture in chloroform solution (0.03 mM). The organic solvent was removed under vacuum and nitrogen to form a dry lipid film, which was then rehydrated with 2 ml PBS containing 60 mg/ml sodium deoxycholate (cholate, Sigma-Aldrich/Merck). This suspension was incubated at 37°C for 30 min until a clear solution containing DMPC/free cholesterol/cholate mixed micelles was obtained. For the preparation of synthetic rHDL-APOE3 and rHDL-APOE4 nanoparticles, mixed micelles were incubated with recombinant APOE3 or APOE4 (Thermo Fisher Scientific Inc) at 59:7:1 DMPC/free cholesterol/APOE molar ratio. Next, three incubation cycles were performed at 4°C and 37°C to promote lipid-protein interaction. After incubation, self-assembly of the synthetic rHDL-APOE nanoparticles started with the removal of cholate through extensive dialysis against a 1000-fold excess of PBS at 4°C for 48 h, using 3,5 kDa Slide-A-Lyzer™ G3 Dialysis Cassettes with two buffer changes. Finally, the dialyzed samples were centrifuged at 16,000 g for 30 min at 4°C to eliminate unbound lipids.

The composition of synthetic rHDL-APOE3 and rHDL-APOE4 nanoparticles, including unesterified cholesterol and APOE, was determined using enzymatic and immunoturbidimetric assays, respectively, with commercial kits adapted for a COBAS 6000 autoanalyzer (Roche Diagnostics and Randox). rHDL-APOE nanoparticles, along with lipid-free APOA1, APOE3, and APOE4 were size-separated electrophoretically on a 4%–15% Mini-PROTEAN® TGX™ Precast Protein Gel (Bio-Rad), followed by protein staining as previously described. Furthermore, particle-size distributions were determined using a dynamic light scattering analyzer together with noninvasive backscatter technology (Malvern Zetasizer, Malvern Instruments). The morphology of synthetic rHDL-APOE3 and rHDL-APOE4 nanoparticles was also observed by transmission electron microscopy with negative staining. An 8 μl aliquot of synthetic rHDL-APOE was added to freshly glow-discharged carbon 300 Mesh copper grids (Ted Pella Inc) for 1 min. After blotting excess fluid, samples were stained with 8 μl of 5% uranyl acetate for 1 min and examined in a JEM 1400 Transmission Electron Microscope (JEOL USA, Peabody).

### Confocal microscopy and flow cytometry

SH-SY5Y neurons were seeded in individual plates of 3.5 cm^2^ (Ibidi®) at a cell density of 50,000 cells per well and differentiated as previously described. Subsequently, 1 ml of serum-free medium with or without synthetic rHDL-APOE3 or rHDL-APOE4 (5 μg/ml), was added to each plate and incubated for 4 h to facilitate the internalization of synthetic rHDL-APOE nanoparticles. The cells were stained with a solution of 0.5 μl of Hoechst and 1 μl of CellMask™ (Thermo Fisher Scientific) diluted in 1 ml of PBS. Imaging of SH-SY5Y neurons was conducted using a Leica TCS SP5 X Tune confocal microscope (Leica).

The uptake of synthetic rHDL-APOE3 and rHDP-APOE4 nanoparticles labeled with Oregon Green™ 488 DHPE fluorophore by SH-SY5Y cells was analyzed using flow cytometry. SH-SY5Y cells were seeded at a density of 200,000 cells per well in 12-well plates, and the differentiation protocol was performed. Upon reaching differentiation, the cells were incubated with or without synthetic rHDL-APOE3 or rHDL-APOE4 (5 μg/ml) in serum-free medium for 4 h. After incubation, cells were washed with PBS, trypsinized for 5 min at 37°C and resuspended in 100 μl of PBS. Flow cytometry analysis was performed using a MACSQuant Analyzer (Miltenyi Biotec), data were acquired for 10,000 events within the gate representing viable single cells and analyzed using MACSQuant Software.

### Statistical analysis

The number of CSF samples per group was estimated using an α value of 0.05, a power of 80%, and an effect size of 1.28 in the cholesterol efflux and uptake assays. The Shapiro-Wilk normality test was conducted to assess Gaussian distribution. Continuous variables are presented as mean ± SD, while qualitative data are expressed as percentages and analyzed using Fisher’s exact test. The Student’s *t* test was used to compare statistical differences between AD and control groups. One-way ANOVA, followed by a post test for linear trend, was used to evaluate relative cholesterol uptake in human neuroblastoma cells, mediated by control CSF lipoproteins, in the absence or presence of t-Tau or Aβ_1-42_. Associations between variables were assessed using Pearson's correlation coefficient. The statistical analyses were conducted using GraphPad Prism 8 (GraphPad, San Diego, CA). Differential expression analysis for proteomics data was performed using limma moderated t-statistics. A *P* value of less than 0.05 was considered statistically significant.

## Results

### CSF cholesterol and APO levels are similar in patients with AD and control individuals

CSF AD biomarkers and CSF cholesterol and APO levels are shown in Table 1. As expected, AD patients had significantly higher CSF concentrations of t-Tau and p-Tau, as well as decreased levels of Aβ_1-42_ and a reduced Aβ_1-42/Aβ1-40_ ratio compared to control individuals. Regarding CSF total and free cholesterol, APO (APOE, APOJ, and APOA1), and PCSK9 concentrations, no significant differences were observed between the groups. The frequency of APOE4 carriers was significantly higher among AD patients (7 APOE-ε4/ε3, 1 APOE-ε4/ε4, and 3 APOE-ε3/ε3), whereas all control individuals were APOE-ε3/ε3. APOE levels did not differ significantly between male and female AD patients (*P* = 0.41).

**Table 1:** Population summary and CSF parameters from control individuals (n = 10) and patients with AD (n = 10)

	Control (n = 10)	AD (n = 10)	*P*
Biological sex (M/F)	8/2	4/6	-
Age (years)	68.6 ± 3.1	72.3 ± 2.7	0.0108
APOE-ϵ4 carriers	0/10	7/10	0.0031
CSF parameters			
Aβ_1-42_ (pg/ml)	1,198.0 ± 330.2	479.1 ± 117.1	<0.0001
Aβ_1-40_ (pg/ml)	11,762.4 ± 2,730.6	11,041.3 ± 2,615.8	0.5540
Aβ_1-42_/Aβ_1-40_ ratio	0.10 ± 0.01	0.04 ± 0.01	<0.0001
t-Tau (pg/ml)	258.5 ± 52.3	763.4 ± 429.82	0.0017
p-Tau (pg/ml)	36.3 ± 6.7	121.15 ± 62.8	0.0005
Total cholesterol (μg/ml)	3.23 ± 0.71	3.21 ± 1.27	0.7936
Free cholesterol (μg/ml)	1.63 ± 0.35	1.67 ± 0.83	0.9116
APOA-I (μg/ml)	0.60 ± 1.01	0.34 ± 0.52	0.4763
APOE (μg/ml)	17.3 ± 9.2	16.5 ± 7.2	0.8305
APOJ (μg/ml)	10.1 ± 2.2	10.7 ± 4.0	0.6662
PCSK9 (ng/ml)	3.23 ± 1.08	3.18 ± 1.56	0.9423

Values are n or mean ± SD. The Shapiro-Wilk normality test was conducted to assess Gaussian distribution. Unpaired *t-tests* were performed for all parameters. Qualitative data are expressed as percentages and analyzed using Fisher’s exact test.

Aβ, amyloid beta; AD, Alzheimer’s disease; APO, apolipoprotein; CSF, cerebrospinal fluid; p-Tau, phosphorylated-Tau181; PCSK9, proprotein convertase subtilisin/kexin type 9.

### Astrocyte cholesterol efflux to CSF is similar in patients with AD and control individuals

**Fig. 1:**
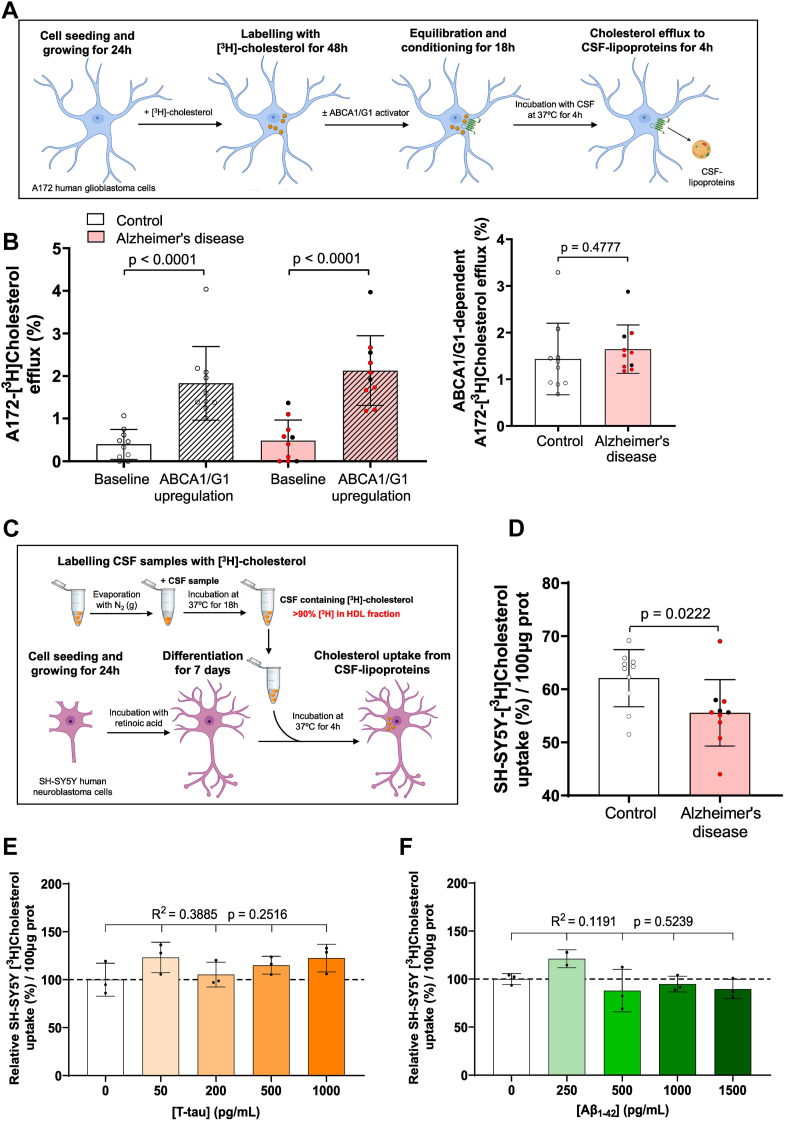
Astrocyte cholesterol efflux to CSF remains similar in AD and control groups, whereas CSF HDL-like-mediated cholesterol delivery to neurons is impaired in AD. (A) Astrocyte cholesterol efflux assay. Human glioblastoma astrocytes were cultured for 24 h, followed by a 48-h incubation with radiolabeled cholesterol. Cells were then treated for 18 h with or without T0901317 to activate ABCA1/G1 pathways. Serum-free medium containing CSF was added for 4 h. Both the medium and cell fractions were processed to quantify radiolabeled cholesterol. (B) Astrocyte cholesterol efflux results: Left panel-Cholesterol efflux from human glioblastoma astrocytes to CSF (30% v/v) is shown for both control and AD samples, under baseline conditions and following T0901317 pretreatment. Right panel-specific ABCA1/G1-dependent cholesterol efflux was calculated subtracting baseline levels from those observed in ABCA1/G1-expressing cells. (C) CSF HDL-like-mediated cholesterol uptake assay: Human neuroblastoma cells were seeded and differentiated into neurons in a low-serum medium containing retinoic acid. After 24 h, radiolabeled CSF HDL-like particles containing unesterified cholesterol were added (10% v/v). The cells were incubated with serum-free medium containing CSF for 4 h, and both the medium and cell lysates were processed for radiolabeled cholesterol quantification. (D) Cholesterol uptake results: CSF HDL-like-mediated cholesterol uptake was measured in human neurons exposed to CSF from both control individuals and patients with AD. Among AD subjects, APOE4 carriers are indicated with a different color. (E and F) Influence of Tau and Aβ_1-42_ on neuronal cholesterol uptake: Cholesterol uptake in SH-SY5Y neurons mediated by control CSF HDL-like particles was assessed in the presence of Tau or Aβ_1-42_ added to the culture media at concentrations up to 1,000 and 1,500 pg/ml, respectively. Values are shown as the mean ± SD for 10 subjects per group in panels (B) and (D) The Student’s *t* test was used to compare the CSF HDL-like-mediated cholesterol uptake by neurons, as well as the astrocyte cholesterol efflux under various conditions between the control and AD groups. One-way ANOVA with a post test for linear trend was used in panels (E) and (F). Three separate experiments were carried out for each condition. Aβ, amyloid beta; AD, Alzheimer’s disease; APO, apolipoprotein; CSF, cerebrospinal fluid.

We adapted a cholesterol efflux assay by loading both A172 human glioblastoma astrocytes and differentiated SH-SY5Y human neurons with radiolabeled unesterified cholesterol and evaluated the release rate of radiolabeled cholesterol to CSF (see Fig. 1A for a schematic diagram of the method involving astrocytes). Cells were also treated with an LXR agonist to compare their responses to ABCA1/G1-mediated cholesterol efflux induction. Under baseline conditions, both cell lines exhibited low cholesterol efflux percentages that were similar to control CSF. However, LXR agonist treatment significantly increased cholesterol efflux in astrocytes, while it remained unchanged in neurons (Supplemental Fig. S1A). Consistent with the cholesterol efflux assay results, both ABCA1 and ABCG1 gene expression were highly upregulated upon LXR agonist exposure in astrocytes. In contrast, these changes were less pronounced in neurons (Supplemental Fig. S1B, C). In addition, we included lipid-free APOA1 and APOE as positive controls in our astrocyte assays to validate cholesterol efflux. Cholesterol efflux from astrocytes was significantly enhanced upon LXR agonist treatment (Supplemental Fig. S2).

Next, we evaluated cholesterol efflux from astrocytes to CSF samples obtained from AD patients and compared the percentage of efflux with that of control individuals under baseline conditions and after pretreating the cells with an LXR agonist. The LXR agonist induced an increase in CSF-mediated cholesterol efflux compared to baseline conditions (Fig. 1B left panel). However, CSF-mediated cholesterol efflux was similar when induced by CSF samples from both patients with AD and control individuals, under baseline conditions and after ABCA1/G1 activation. Furthermore, when comparing ABCA1/G1-dependent cholesterol efflux between groups (calculated as the increase in efflux after subtracting baseline levels), CSF from AD patients and control individuals induced cholesterol efflux at similar levels (Fig. 1B right panel).

### CSF lipoprotein-mediated cholesterol delivery to neurons is impaired in patients with AD

We developed a cholesterol uptake assay by incorporating radiolabeled unesterified cholesterol into the lipoproteins of CSF and evaluated the rate of radiolabeled cholesterol uptake in both A172 human glioblastoma astrocytes and differentiated SH-SY5Y human neurons (see Fig. 1C for a schematic diagram of the method involving neurons). To establish optimal conditions for cholesterol uptake, neurons were incubated with radiolabeled control CSF for 2, 4, and 8 h. The percentage of CSF-mediated cholesterol uptake in neurons increased linearly in a time-dependent manner (Supplemental Fig. S3A). In contrast, cholesterol uptake assays in astrocytes showed no significant increase in radiolabeled cholesterol uptake over the same time points (Supplemental Fig. S3A), suggesting that neurons may depend more heavily on external cholesterol sources (Supplemental Fig. S3B). To evaluate cholesterol delivery to neurons, we then compared CSF lipoprotein-mediated cholesterol uptake in differentiated SH-SY5Y human neurons from CSF samples acquired from control individuals and patients with AD. The percentage of radiolabeled cholesterol uptake within a 4-h period was significantly reduced in neurons incubated with CSF from AD patients (Fig. 1D). Given that Aβ and Tau may potentially alter the lipid-binding function of CSF lipoproteins (30, 31, 32), we examined the association between CSF-mediated cholesterol uptake and other CSF parameters (Table 2). We observed a positive trend between CSF-mediated cholesterol uptake and CSF Aβ_1-42_ levels; however, this correlation did not reach statistical significance (Table 2). To further investigate whether Aβ_1-42_ or even Tau could directly alter CSF-mediated cholesterol uptake in neurons, we added isolated Aβ_1-42_ or Tau to CSF in the presence of differentiated neurons at increasing concentrations. Neither Tau nor Aβ_1-42_ affected the percentage of radiolabeled cholesterol uptake in neurons within the physiological range (Fig. 1E, F).

**Table 2:** Association of neuronal CSF-mediated cholesterol uptake with CSF biochemical parameters

	Pearson r	*P*
Aβ_1-42_ (pg/ml)	0.4081	0.0741
Aβ_1-40_ (pg/ml)	0.2206	0.3499
Aβ_1-42/Aβ1-40_ ratio	0.4359	0.0547
t-Tau (pg/ml)	−0.0145	0.9516
p-Tau (pg/ml)	−0.0960	0.6871
Total cholesterol (μg/ml)	−0.1300	0.5847
Free cholesterol (μg/ml)	0.2029	0.3908
APOA-I (μg/ml)	0.0601	0.8014
APOE (μg/ml)	−0.1484	0.5322
APOJ (μg/ml)	0.1086	0.6485
PCSK9 (ng/ml)	−0.1105	0.6429

Associations between variables were assessed using Pearson's correlation coefficient.

Aβ, amyloid beta; CSF, cerebrospinal fluid; p-Tau, phosphorylated-Tau181; PCSK9, proprotein convertase subtilisin/kexin type 9.

Furthermore, CSF-mediated cholesterol uptake was not associated with age in either control individuals (Pearson r = 0.37, *P* = 0.29) or AD patients (Pearson r = −0.10, *P* = 0.66). In addition, no significant differences were observed between males and females (*P* = 0.70) in the AD group, where the sex distribution was balanced.

### The CSF lipoprotein proteome is modified in patients with AD

**Fig. 2:**
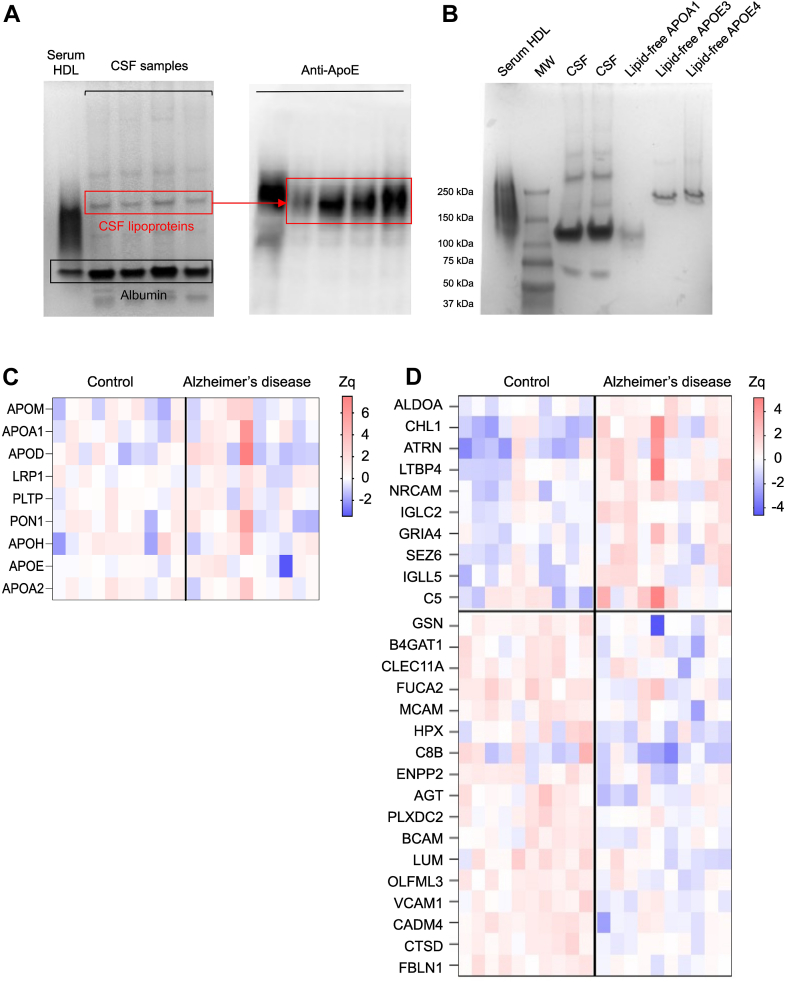
Altered proteome of CSF HDL-like particles in AD. (A) Native Gel Electrophoresis of CSF samples. Representative native polyacrylamide gel electrophoresis of CSF from patients with AD and control individuals. Left panel-Coomassie blue staining showing lipoprotein and albumin bands. Right panel-corresponding nitrocellulose blot probed with a polyclonal anti-APOE antibody, identifying the APOE-containing band using nondenaturing polyacrylamide gradient gel electrophoresis. (B) Native gel electrophoresis of isolated serum HDL, CSF samples, lipid-free APOA1, lipid-free APOE3 and lipid-free APOE4. (C) Proteins involved in cholesterol metabolism quantified in the APOE-containing lipoprotein band from CSF. Proteins with increased (red) or decreased (blue) abundance in AD versus control are shown according to the indicated Zq score scale. APOM: apolipoprotein M; APOA1: apolipoprotein A-I; APOD: apolipoprotein D; LRP1: low-density lipoprotein receptor-related protein 1; PLTP: phospholipid transfer protein; PON1: serum paraoxonase/arylesterase 1; APOH: Beta-2-glycoprotein 1; APOE: apolipoprotein E; APOA2: apolipoprotein A-II. (D) Differentially regulated proteins in HDL-like particles from the CSF of patients with AD. Heat map depicting significant protein abundance changes (*P* < 0.05, proteins quantified with more than one single peptide) in AD and control groups (10 independent samples per group). Increased (red) or decreased (blue) abundances are shown according to the indicated Zq scale. Differential protein expression analysis was performed using moderated t-statistics (limma test). ALDOA: fructose-bisphosphate aldolase A; ACHL1: neural cell adhesion molecule L1-like protein; ATRN: attractin; LTBP4: latent-transforming growth factor beta-binding protein 4; NrCAM: neuronal cell adhesion molecule; IGLC2: immunoglobulin lambda constant 2; GRIA4: glutamate receptor 4; SEZ6: seizure protein 6 homolog; IGLL5: immunoglobulin lambda-like polypeptide 5; C5: complement C5; GELS: gelsolin; B4GAT1: beta-1,4-glucoronyltransferase 1; CLEC11A: C-type lectin domain family 11 member A; FUCA2: plasma alpha-L-fucosidase; MCAM: cell surface glycoprotein MUC18; HPX: hemopexin; C8B: complement component C8 beta chain; ENPP2: ectonucleotide pyrophosphatase/phosphodiesterase family member 2; AGT: angiotensinogen; PLXDC2: plexin domain-containing protein 2; BCAM: basal cell adhesion molecule; LUM: lumican; OLFML3: isoform 2 of olfactomedin-like protein 3; VCAM1: vascular cell adhesion protein 1; CADM4: cell adhesion molecule 4; CTSD: cathepsin D; FBLN1: fibulin-1; AD, Alzheimer’s disease; APO, apolipoprotein; CSF, cerebrospinal fluid.

We separated CSF lipoproteins according to size using nondenaturing polyacrylamide gradient gel electrophoresis. Native gels revealed a homogeneous band corresponding to large HDL particles (Fig. 2A, left panel). Western blot analysis confirmed that APOE was localized at the same position as the lipoprotein band (Fig. 2A, right panel). To further characterize APOE migration, we ran lipid-free APOE3 and APOE4 in parallel with CSF samples (Fig. 2B). Although lipid-free APOE can form multimers and migrate above its apparent molecular weight—similar to albumin and lipid-free APOA1—we observed distinct migration patterns between lipid-free APOE isoforms and APOE-containing lipoproteins in CSF under native conditions (Fig. 2B).

Next, we separated the CSF lipoproteins from the CSF of patients with AD and control individuals with the polyacrylamide gradient gel electrophoresis, and performed a comparative MS profiling of proteins differentially expressed in the APOE-containing particles band. A total of 239 proteins were detected with two or more peptides and were consistently present across all CSF samples (Supplemental Table S1). Nine proteins were related to cholesterol metabolism, among which APOE was the most abundant protein. However, none of the cholesterol-related proteins showed significant differences between the control and AD groups (Fig. 2B). In contrast, the relative abundance of 27 noncholesterol-related proteins changed significantly in patients with AD compared to controls, with 10 upregulated and 17 downregulated (Fig. 2C). Functional enrichment analysis revealed that the majority of these proteins were mainly involved in apoptotic cell clearance, fibrinolysis, acute-phase response, complement activation, regulation of immune and inflammatory responses, cell adhesion, and proteolysis (Supplemental Fig. S4).

### APOE4-containing HDL exhibits impaired cholesterol delivery to neurons

**Fig. 3:**
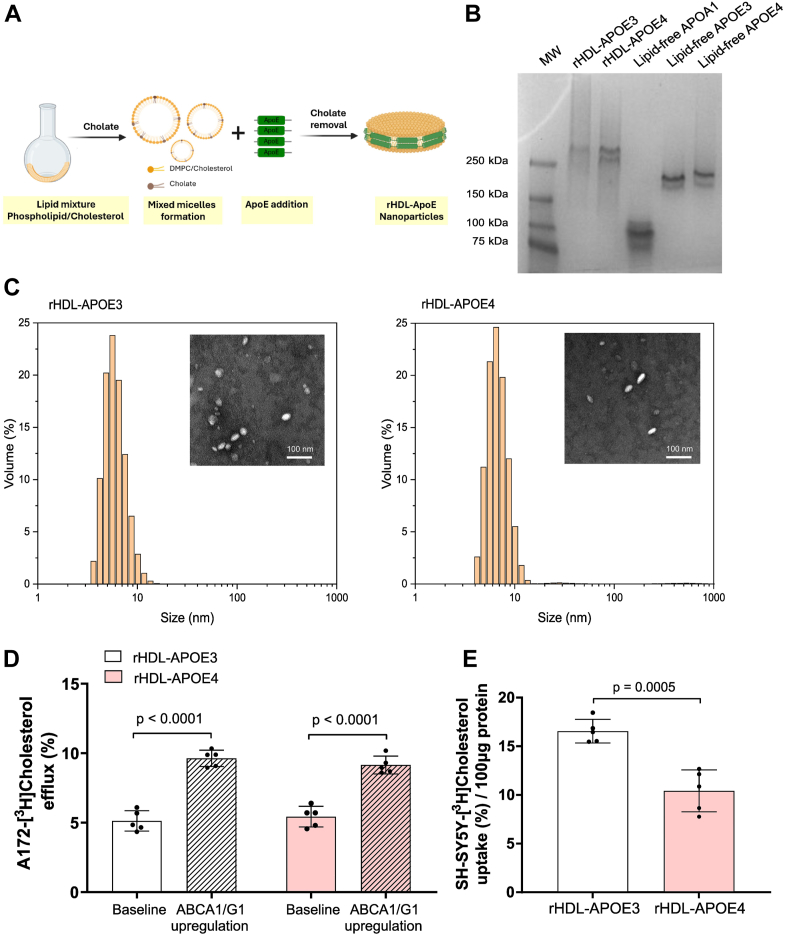
Synthetic rHDL nanoparticles containing APOE4 exhibit impaired cholesterol delivery to neurons. (A) Schematic representation of the synthesis of rHDL-APOE nanoparticles: recombinant APOE3 or APOE4 were combined with DMPC and cholesterol in a molar ratio of 59:7:1. The mixture underwent three cycles of vortexing and temperature modulation, alternating between 37°C and 4°C, to optimize APOE-lipid interactions. (B) Native gel electrophoresis of synthetic rHDL-APOE3 and HDL-APOE4 nanoparticles: Representative native polyacrylamide gel electrophoresis image of synthetic rHDL-APOE3 and rHDL-APOE4 nanoparticles, lipid-free APOA1, lipid-free APOE3, and lipid-free APOE4, visualized with Coomassie Blue staining. (C) Characterization of synthetic rHDL-APOE3 and HDL-APOE4 nanoparticles: The particle size distributions of purified synthetic rHDL-APOE3 and rHDL-APOE4 nanoparticles were analyzed using dynamic light scattering. Representative images from also shown as insets. (D) Astrocyte cholesterol efflux to synthetic rHDL-APOE3 and rHDL-APOE4: Cholesterol efflux from human glioblastoma astrocytes to synthetic rHDL-APOE3 and rHDL-APOE4 (5 μg/ml) was measured under baseline conditions and after T0901317 pretreatment, as described in Fig. 1A. (E) Neuronal cholesterol uptake mediated by synthetic rHDL-APOE3 and rHDL-APOE4: synthetic rHDL-APOE3 and rHDL-APOE4 (5 μg/ml) were loaded with radiolabeled unesterified cholesterol, and their capacity to facilitate cholesterol uptake in human neuroblastoma cells was assessed as described in Fig. 1C. Mean ± SD is used to express values. Student t-tests were used to compare HDL-mediated neuronal cholesterol delivery between synthetic rHDL-APOE3 and rHDL-APOE4, as well as astrocyte cholesterol efflux under various conditions. Five separate experiments were conducted to evaluate each condition. APO, apolipoprotein; DMPC: 1,2-dimyristoyl-sn-glycero-3-phosphocoline; rHDL, reconstituted HDL.

Given the higher prevalence of APOE4 carriers among patients with AD, we prepared both synthetic rHDL-APOE3 and HDL-APOE4 nanoparticles by assembling phospholipids and cholesterol with recombinant human APOE3 or APOE4 (see Fig. 3A for a schematic diagram of their synthesis). Both synthetic rHDL-APOE3 and rHDL-APOE4 nanoparticles showed a similar HDL migration pattern (Fig. 3B) and size (Fig. 3C), as well as a similar cholesterol/APOE ratio (0.278 for synthetic rHDL-APOE3 and 0.287 for rHDL-APOE4).

**Fig. 4:**
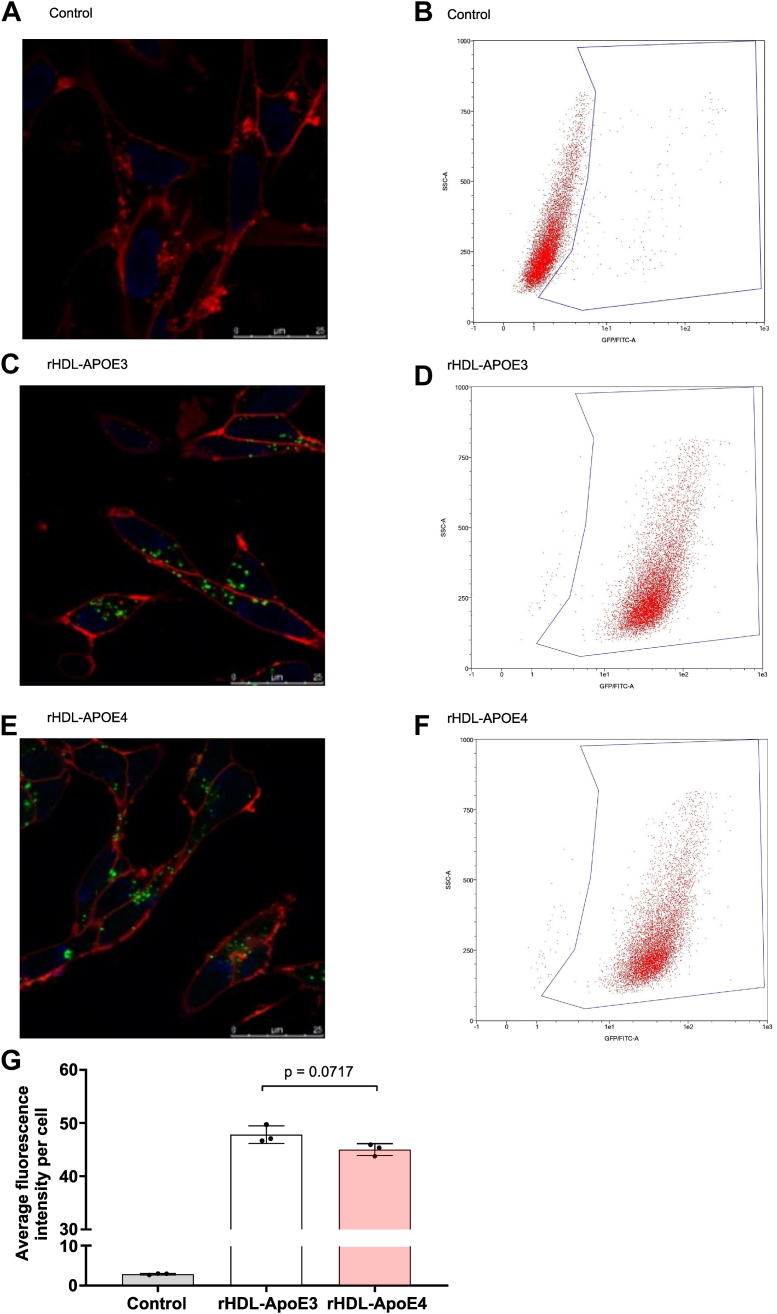
Reconstituted Oregon Green 488-Labeled HDL-APOE nanoparticles internalization in human neuroblastoma cells. The phospholipid 1,2-dihexadecanoyl-sn-glycero-3-phosphoethanolamine was labeled with Oregon Green 488 and incorporated into synthetic rHDL-APOE3 and HDL-APOE4 nanoparticles. Human neuroblastoma cells were incubated with the labeled nanoparticles or left untreated as a control for 4 h, followed by confocal microscopy and flow cytometry analyses. Representative images of SH-SY5Y neurons and scatter plots showing side scatter (SSC-A) plotted against green fluorescence (GFP/FITC-A): (A and B) Representative control cells (not labeled); (C and D) Cells treated with synthetic rHDL-APOE3 nanoparticles; (E and F) Cells treated with synthetic rHDL-APOE4 nanoparticles. (G) The graph displays fluorescence phospholipid uptake into cells based on data obtained from flow cytometry. Average fluorescence intensity per cell is shown, representing the degree of nanoparticle uptake. Student *t* test was used to compare both groups. Three independent experiments were conducted to evaluate each condition. APO, apolipoprotein; rHDL, reconstituted HDL.

We then mimicked cholesterol efflux from astrocytes by replacing the AD and control CSFs with synthetic rHDL-APOE3 and rHDL-APOE4 nanoparticles, following the method depicted in Figure 1A. When comparing cholesterol efflux between the two rHDL nanoparticles, both synthetic rHDL-APOE3 and rHDL-APOE4 induced cholesterol efflux from astrocytes at similar levels. (Fig. 3D). We also compared the ability of both synthetic rHDLs, which were radiolabeled as in the graphic depicted in Figure 1C, to mediate cholesterol uptake in neurons. The percentage of radiolabeled cholesterol uptake was significantly reduced in neurons incubated with synthetic rHDL-APOE4 compared to rHDL-APOE3 (Fig. 3E). We further evaluated the capacity of neurons to internalize an Oregon Green–labeled phospholipid incorporated into synthetic rHDL-APOE3 and rHDL-APOE4 nanoparticles using confocal microscopy and a flow cytometry assay (Fig. 4A–F). After a 4-h incubation with the fluorescently labeled nanoparticles, SH-SY5Y neurons exhibited a trend toward decreased internalization of rHDL-APOE4-associated phospholipids compared with rHDL-APOE3 (Fig. 4G).

## Discussion

In this study, we aimed to assess the ability of lipoproteins from AD patients’ CSF to promote cholesterol efflux from human glioblastoma astrocytes and evaluate their capacity to deliver cholesterol to human neurons. Previous studies have reported mixed results regarding APOE levels in AD (18, 19, 33, 34, 35, 36, 37, 38). In our study, CSF APOE levels were similar between the AD and control groups and were unaffected by sex distribution. In addition, the levels of CSF APOA1 and cholesterol were similar between patients with AD and control subjects. Thus, within the limits of our sample size and variability, we did not observe significant differences in the main CSF lipoprotein lipid and protein components between AD patients with or without the APOE4 allele. Astrocyte cholesterol efflux to CSF lipoproteins containing APOE is a complex process involving both passive diffusion—driven by concentration gradient—and active energy-dependent pathways mediated by ABCA1 and ABCG1 transporters (16, 39). We evaluated the ability of human A172 glioblastoma astrocytes and SH-SY5Y neurons to mediate cholesterol efflux to CSF under basal conditions and following stimulation of the ABCA1-and ABCG1-dependent pathways. Consistent with previous findings (39), human A172 glioblastoma astrocytes showed a higher capacity for inducing ABCA1/G1-mediated cholesterol efflux to CSF compared to neurons. Notably, astrocyte cholesterol efflux rates to CSF were similar between AD and control groups, both under baseline conditions and after activating of the ABCA1/G1-dependent pathway. This outcome aligns with the findings of an earlier report (33), which observed comparable cholesterol efflux to CSF from rat astrocytes incubated under baseline conditions in both AD and control samples. Conversely, another study found reduced cholesterol efflux from human glioblastoma U373-MG astrocytoma cells to AD CSF compared to control CSF after ABCA1/G1-dependent pathway stimulation (40). The higher CSF APOE levels in AD patients in this latter study might explain the reduced capacity of AD CSF to promote astrocyte cholesterol efflux, similar to the effect observed with macrophages (41). It should be noted that human astrocyte models with the APOE4 genotype have demonstrated reduced cholesterol efflux to plasma HDL, primarily due to APOE4’s negative impact on ABCA1 expression at the cell membrane (17, 42). Indeed, human induced pluripotent stem cell-derived astrocytes secrete abundant APOE lipoprotein particles, with APOE4 particles transporting fewer lipids compared to APOE3 particles (13). In our study, however, we used human glioblastoma astrocytes with identical genetic material to specifically assess potential differences in the ability of CSF lipoproteins to promote cellular cholesterol efflux, thereby minimizing any confounding effects from cellular APOE variations.

To study cholesterol delivery, we developed an isotopic cholesterol uptake assay by incorporating radiolabeled unesterified cholesterol into the lipoproteins of CSF to evaluate the ability of human A172 glioblastoma astrocytes and SH-SY5Y neurons to take up cholesterol from CSF. The high cholesterol uptake rates observed in human SH-SY5Y neurons confirmed their suitability as a model for cholesterol uptake assays. Our results also revealed that, compared to controls, cholesterol uptake in human neurons was significantly reduced when mediated by CSF from patients with AD, indicating a diminished capacity of neurons to acquire cholesterol from lipoprotein particles in AD CSF. Although CSF levels of PCSK9—the primary protein responsible for degrading APOE-binding receptors—have been reported as elevated in patients with AD in one study (43), we observed no differences in CSF PCSK9 concentrations between AD and control groups. Thus, the role of PCSK9 in AD remains unclear and warrants further investigation.

Although several studies have investigated the protein composition of CSF, only one recent study has specifically analyzed the lipoprotein proteome in CSF from control individuals, utilizing fluorescent high-resolution size-exclusion chromatography fractionation (44)—though this required a substantial CSF volume—. In our study, we characterized the proteome of APOE-containing band with size slightly larger than plasma HDL in CSF samples from both patients with AD and control individuals. We compared our proteomic dataset with the lipoprotein proteome derived from 350 μl of pooled CSF using fluorescent profiling and high-sensitivity LC-MS/MS (44). Despite the use of lower CSF volumes and a distinct gel-based isolation approach in our study, 52.9% of the proteins quantified in our APOE-enriched particles overlapped with those identified in the published dataset. With our approach, we identified a total of 239 proteins, consistently present across all CSF samples. Regarding cholesterol metabolism-related proteins, nine were consistently present in all CSF samples, with no significant quantitative differences between AD and control groups. The lack of differences in CSF lipoprotein-associated APOE levels aligns with our CSF biochemical analyses and those of Martinez-Morillo *et al.* (45), who also observed no difference in CSF APOE levels between patients with AD and non-AD patients when measured by mass spectrometry. We also identified 27 noncholesterol metabolism-related proteins that were expressed differently in AD and control groups. Among the proteins upregulated in the APOE-containing band from patients with AD, aldolase A (ALDOA) and neuronal cell adhesion molecule (NrCAM) are particularly noteworthy. ALDOA, a glycolytic enzyme, is elevated in the CSF of patients with AD and has been linked to impaired glucose metabolism in the AD brain (46). NrCAM, a synaptic cell adhesion molecule, is crucial for synaptic plasticity and has been implicated in AD pathology via its interactions with Aβ and its impact on synapse function and integrity (47). Among the downregulated proteins, fibulin-1 (FBLN1) and cathepsin D (CTSD) are of particular interest. FBLN1 binds to the N-terminal domain of APP, modulating its neurotrophic activity (48), while CTSD, a lysosomal protease, plays a role in the autophagy-lysosomal Aβ clearance pathway (49). It remains unclear whether the association of these proteins with lipoprotein particles in CSF affects their functions in neuroinflammation, complement activation, neurotrophic activity, and amyloid-beta toxicity, warranting further investigation into their potential roles.

Since our biochemical and proteomic analyses revealed no differences in CSF lipoproteins involved in cholesterol transport, we investigated whether the observed alterations in cholesterol transport could arise from functional differences among the APOE isoforms. Previous studies have reported conflicting findings on the lipidation of APOE isoforms in astrocytes. One study observed similar ABCA1-dependent cholesterol and phosphatidylcholine efflux from astrocytes to recombinant lipid-free APOE regardless of the APOE isoform (50), while another study found that recombinant lipid-free APOE3 facilitated greater cholesterol efflux compared to APOE4 (51). These latter findings align with the lower ABCA1 expression and reduced cholesterol efflux observed in rodent primary astrocytes incubated with recombinant APOE4 (17). In our study, we directly evaluated the ability of synthetic rHDL nanoparticles containing either recombinant APOE3 or APOE4 isoforms to promote both cholesterol efflux from human A172 glioblastoma astrocytes and cholesterol uptake by SH-SY5Y neurons. These rHDL-APOE particles, which were lipidated with cholesterol and phospholipids, formed particles larger than lipid-free APOE isoforms. Although structural differences may influence uptake dynamics, the use of reconstituted particles enabled a controlled comparison of APOE isoforms, minimizing the variability inherent in heterogeneous human lipoproteins and genotypes. Our results showed no significant differences in cholesterol efflux between synthetic rHDL-APOE3 and HDL-APOE4. However, in the cholesterol uptake assay, SH-SY5Y neurons exhibited a significantly reduced capacity to internalize radiolabeled cholesterol when mediated by synthetic rHDL-APOE4 compared to rHDL-APOE3. This suggests that the APOE4 isoform may have a lower affinity or weaker interaction with neuronal APOE-binding receptors, impairing neuronal cholesterol delivery. Supporting these findings, a previous study using hippocampal rat neurons also demonstrated that cholesterol uptake is dependent on the APOE isoform, with reduced radiolabeled cholesterol delivery to rat neurons when bound to lipidated APOE4 isoform (52).

To further contextualize this, APOE is known to interact with multiple receptors in the LDLR family, including LDLR, LRP1, very-low density lipoprotein receptor, and APOE receptor 2, which are expressed in both peripheral tissues and the CNS, particularly in neurons (53). Among these, LRP1 exhibits especially high transport capacity due to its rapid endocytic recycling (54). Notably, selective deletion of LRP1 in forebrain neurons leads to widespread lipid metabolic dysfunction and neurodegeneration (55), underscoring the critical role of receptor-mediated lipid uptake in brain homeostasis. While the rHDL-APOE particles used in our study are structurally simpler than native CSF lipoproteins, they are still likely to engage these physiologically relevant receptors. Thus, our model provides a useful tool for probing isoform-specific differences in neuronal cholesterol delivery and supports a mechanistic link between APOE4 and impaired lipid metabolism in the CNS.

Although we anticipated that neuronal phospholipid uptake would mirror the APOE isoform-dependent differences observed in cholesterol uptake, our results revealed only a modest reduction of phospholipids uptake in the presence of rHDL-APOE4 particles. This suggests that neuronal lipid uptake is selective and may be differentially regulated across lipid classes. Cholesterol, given its critical role in maintaining membrane integrity and supporting lipid raft formation in neurons, is likely subject to more tightly controlled, receptor-mediated uptake pathways. In contrast, phospholipid uptake may occur through alternative mechanisms, with a greater proportion occurring independently of lipoprotein endocytosis and via distinct intracellular processing routes (56). These findings underscore the specificity of APOE4’s impact on cholesterol transport and highlight the complex regulation of neuronal lipid homeostasis.

As a limitation of this study, we must consider that the cholesterol export activity of glioblastoma astrocytes may be exceptionally high and significantly different from that of astrocytes in situ in the adult or aged brain. Although the statistical power calculations indicated that a sample size of ten per group would provide sufficient sensitivity to detect changes, the sample size limited our ability to compare the APOE-ε4 versus APOE-ε3 genotypes among the AD samples. In addition, the control and AD groups were not perfectly matched for age and sex, with fewer females in the control group. Nevertheless, based on exploratory analyses within the current sample size, variations in age and sex distribution did not appear to influence CSF HDL-like-mediated cholesterol delivery to neurons.

## Conclusions

Our findings indicate that cholesterol transport from CSF lipoproteins into neurons is impaired in AD patients. Biochemical and proteomic analysis of CSF APOE-containing particles showed no significant differences in cholesterol metabolism-related proteins between patients with AD and controls, and neither Tau nor Aβ was found to interfere with this process. Notably, neurons show impaired uptake of APOE4 associated rHDL-like particles compared to APOE3. Future prospective studies are needed to establish whether impaired neuronal cholesterol delivery directly contributes to AD progression.

## Data availability

Data are provided within the manuscript or supplementary information files. The mass spectrometry proteomics data have been deposited to the ProteomeXchange Consortium via the PRIDE partner repository with the dataset identifier PXD057614.

## Supplemental data

This article contains supplemental data.

## Conflict of interest

J. F. reported receiving personal fees for service on the advisory boards, adjudication committees or speaker honoraria from AC Immune, Adamed, Alzheon, Biogen, Eisai, Esteve, Fujirebio, Ionis, Laboratorios Carnot, Life Molecular Imaging, Lilly, Lundbeck, Perha, Roche, Zambón and outside the submitted work. J. F. reports holding a patent for markers of synaptopathy in neurodegenerative disease (licensed to ADx, EPI8382175.0). The other authors declare that they have no conflicts of interest with the contents of this article.
