# Supplementary material for: Cerebrospinal fluid lipoprotein-mediated cholesterol delivery to neurons is impaired in Alzheimer's disease and involves APOE4
Source: J Lipid Res. 2025 Jul 21;66(8):100865. doi: 10.1016/j.jlr.2025.100865 (PMC12391596; doi:10.1016/j.jlr.2025.100865)
Supplement: Supplementary Material [file mmc1.docx]

**Cerebrospinal fluid lipoprotein-mediated cholesterol delivery to neurons is impaired in Alzheimer's disease and involves APOE4**

**Carla Borràs MsC ^1,2,3^, Marina Canyelles PhD ^1,3,4^,** **David Santos BSc ^1,3^, Noemí Rotllan PhD ^1,3^, Estefanía Núñez PhD ^5,6^, Jesús Vázquez PhD ^5,6^, Daniel Maspoch PhD ^7,8,9^, Mary Cano-Sarabia PhD ^7,^**^8^**, Qi Zhao MSc ^1,2^, Maria Carmona-Iragui MD PhD ^1,10,11^, Sònia Sirisi PhD ^1,10,11^, Alberto Lleó MD PhD ^1,10,11^, Juan Fortea MD PhD ^1,10,11,12^, Daniel Alcolea MD PhD ^1,10,11^, Francisco Blanco-Vaca MD PhD ^*,1,2,3,4^, Joan Carles Escolà-Gil PhD ^*,1,2,3,^, Mireia Tondo PhD ^*,1,3,4^**

^1^ Institut de Recerca Sant Pau (IR SANT PAU), Barcelona, Spain.

^2^ Departament de Bioquímica i Biologia Molecular, Universitat Autònoma de Barcelona, Barcelona, Spain.

^3^ CIBER de Diabetes y Enfermedades Metabólicas Asociadas, CIBERDEM, Madrid, Spain.

^4^ Department of Biochemistry, Hospital de la Santa Creu i Sant Pau, Barcelona, Spain.

^5^ Centro Nacional de Investigaciones Cardiovasculares Carlos III, Madrid, Spain.

^6^ CIBER de Enfermedades Cardiovasculares, CIBERCV, Madrid, Spain.

^7^ Catalan Institute of Nanoscience and Nanotechnology (ICN2), CSIC and The Barcelona Institute of Science and Technology, Campus UAB, Bellaterra, Barcelona, Spain.

^8^ Departament de Química, Facultat de Ciències, Universitat Autònoma de Barcelona (UAB), Cerdanyola del Vallès, Barcelona, Spain.

^9^ ICREA (Institució Catalana d’Investigació i Estudis Avançats), Barcelona, Spain.

^10^ Sant Pau Memory Unit, Department of Neurology, Hospital de la Santa Creu i Sant Pau, Barcelona, Spain.

^11^ CIBER de Enfermedades Neurodegenerativas, CIBERNED, Madrid, Spain.

^12^ Barcelona Down Medical Center, Fundació Catalana de Síndrome de Down, Barcelona, Spain

^*^ These authors contributed equally

Address for correspondence: Mireia Tondo ([mtondo@santpau.cat](mailto:mtondo@santpau.cat), tel. +34 93 5537358) and Joan Carles Escolà-Gil ([jescola@santpau.cat](mailto:jescola@santpau.cat), tel. +34 93 5537588) Institut de Recerca Sant Pau, C/ Sant Quintí 77-79, 08041 Barcelona, Spain.

Short title: Impaired Cholesterol Delivery to Neurons in Alzheimer’s disease


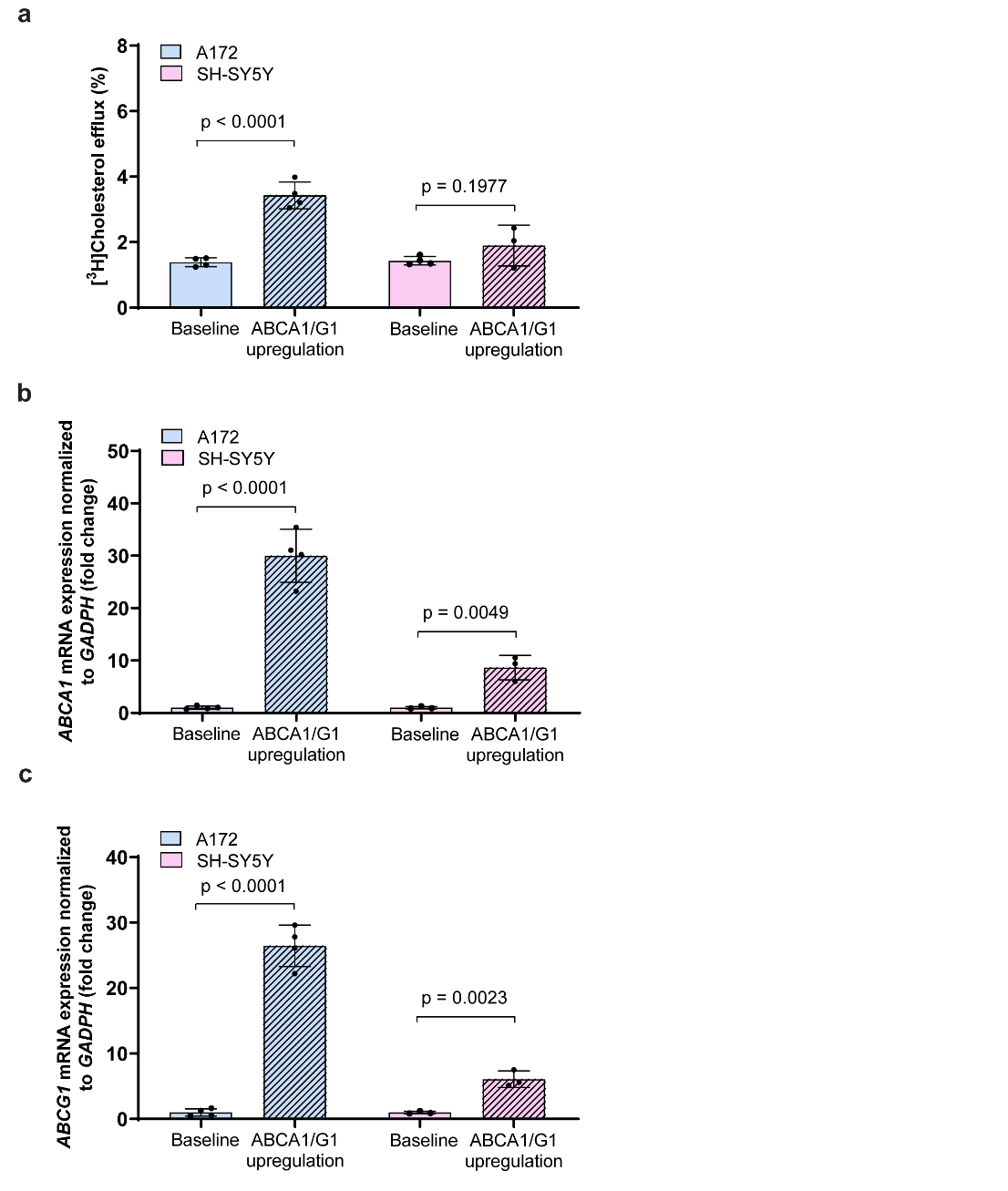


**Supplemental Figure S1. Regulation of ABCA1/G1-Mediated Cholesterol Efflux to CSF in Human Glioblastoma Astrocytes. (a)** **Cholesterol Efflux to Control CSF:** Cholesterol efflux from human glioblastoma astrocytes and human neurons to control CSF (30% v/v) was measured under baseline conditions and after pre-treatment with T0901317. **(b and c):** ***ABCA1* and *ABCG1* mRNA Expression:** Cellular mRNA was extracted, and quantitative real-time PCR was performed to assess relative mRNA expression of *ABCA1* and *ABCG1*, normalized to *GAPDH*. Baseline expression for each cell type was set to 1 arbitrary unit and subsequent expression levels were expressed as fold changes.

Student t-tests were used to compare the efflux of cholesterol between neurons and astrocytes under various conditions. Four independent experiments were conducted to evaluate each condition.

**Supplemental Figure S2. Cholesterol Efflux to** **lipid-free APOA1 and lipid-free APOE is enhanced after ABCA1/G1 pathways are induced.** Cholesterol efflux from human glioblastoma astrocytes to lipid-free APOA1 and lipid-free APOE at a concentration of 20 μg/mL was measured under baseline conditions and after pre-treatment with T0901317.
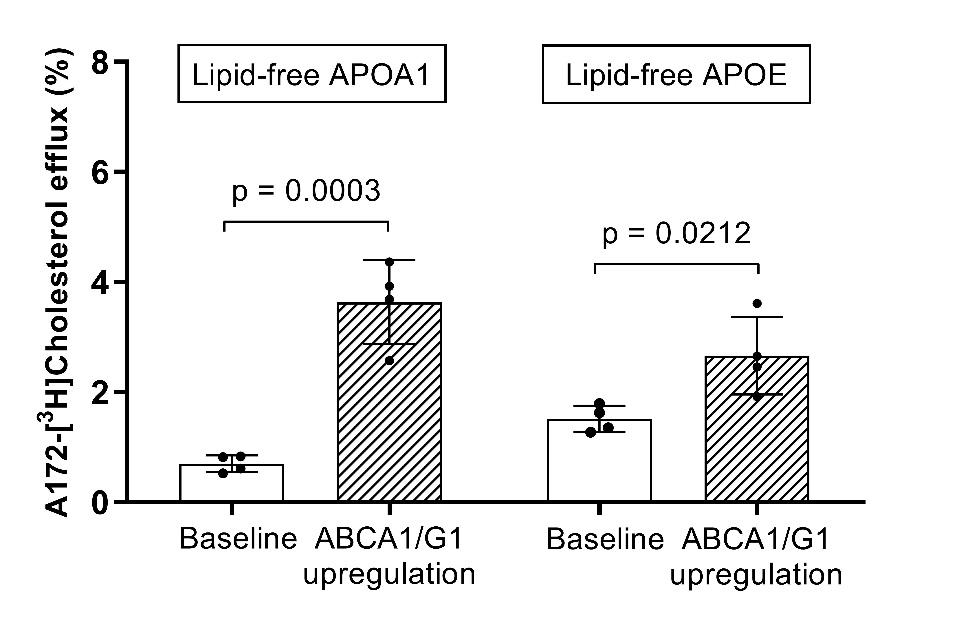


Student t-tests were used to compare the efflux of cholesterol between conditions. Four independent experiments were conducted to evaluate each condition.


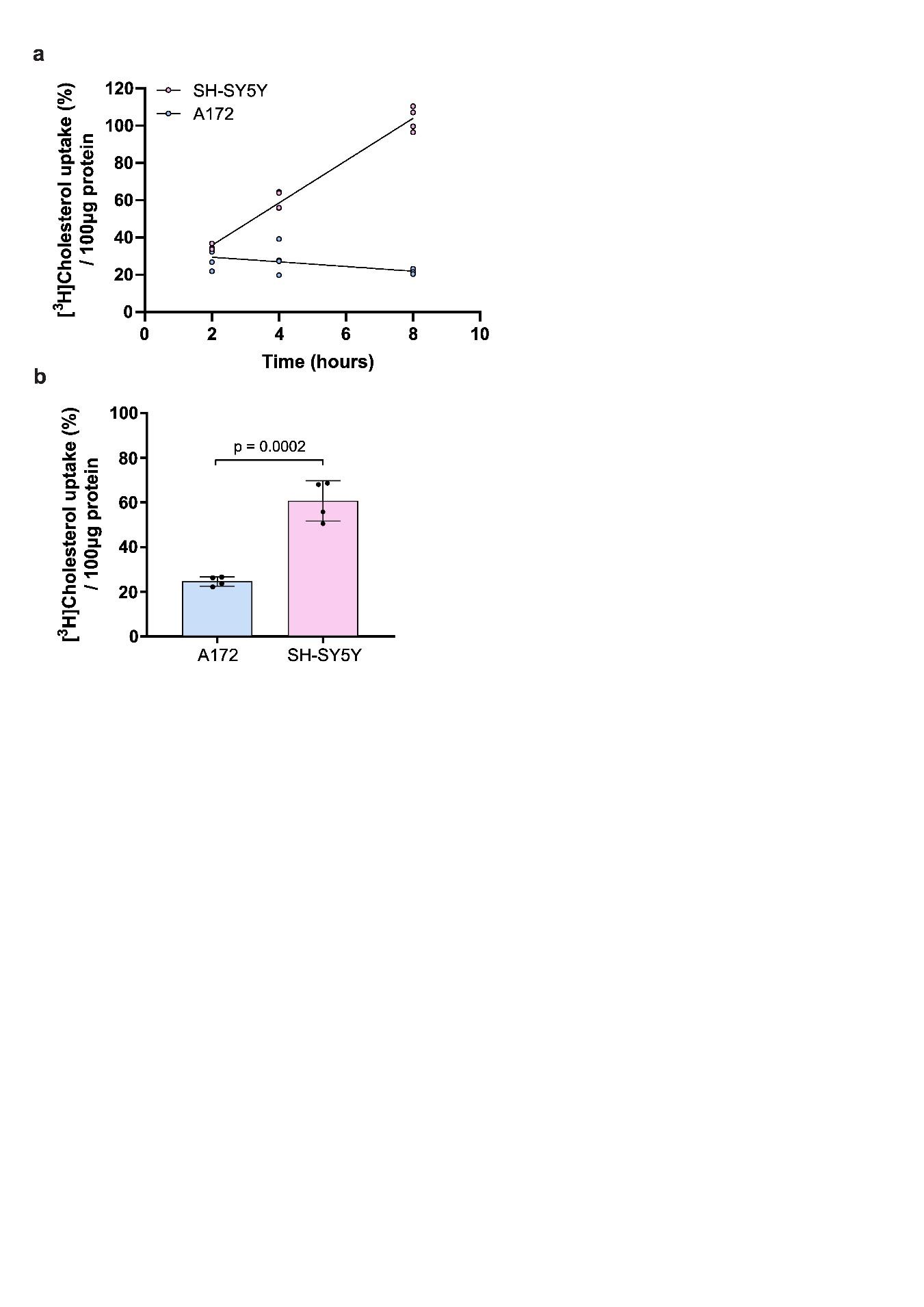


**Supplemental Figure S3. Neurons Exhibit Higher Relative CSF-Mediated Cholesterol Uptake Compared to Astrocytes.** **(a)** **Time-Course Analysis of Cholesterol Uptake:** Control CSF (10% v/v) was used to assess cholesterol uptake over time in human glioblastoma astrocytes and human neurons. **(b)** **Comparison of Cholesterol Uptake Between Cell Types:** The 4-hour control CSF lipoprotein-mediated cholesterol uptake was compared between human glioblastoma astrocytes and human neurons.

A Student t-test was used to evaluate differences in CSF-mediated cholesterol uptake between astrocytes and neurons. Four independent experiments were conducted to evaluate each condition.

**
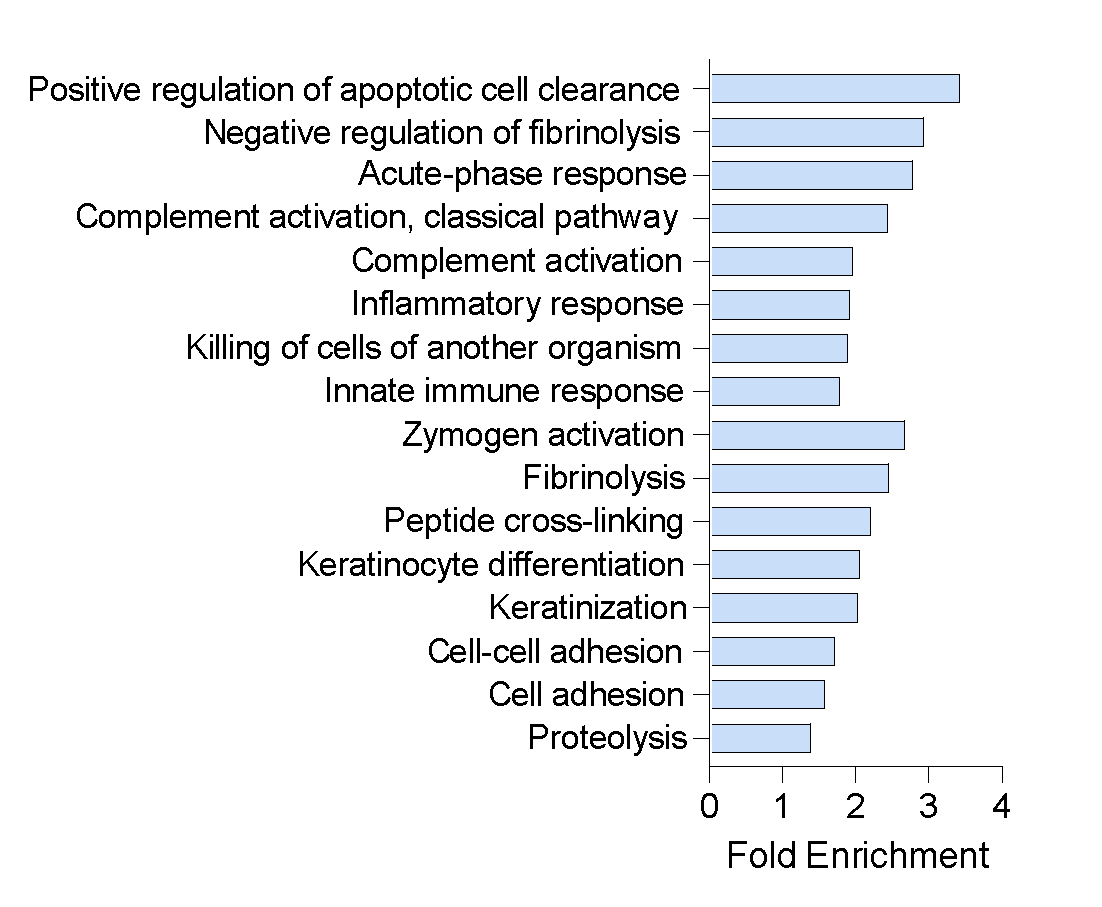
**

**Supplemental Figure S4. Enrichment Analysis of Proteins Quantified in lipoprotein band from CSF.** The bars show the fold enrichment of each of the main biological processes (GOBP categories) that are significantly enriched (EASE score<0.05).
